# Prevalence of Intestinal Parasitoses in a Non-Endemic Setting during a 10-Year Period (2011–2020): A Focus on *Dientamoeba fragilis*

**DOI:** 10.3390/microorganisms10020426

**Published:** 2022-02-12

**Authors:** Adriana Calderaro, Mirko Buttrini, Sara Montecchini, Sabina Rossi, Benedetta Farina, Maria Cristina Arcangeletti, Flora De Conto, Carlo Chezzi

**Affiliations:** 1 Department of Medicine and Surgery, University of Parma, Viale A. Gramsci 14, 43126 Parma, Italy; mirko.buttrini@unipr.it (M.B.); sara.montecchini@unipr.it (S.M.); benedetta.farina@unipr.it (B.F.); mariacristina.arcangeletti@unipr.it (M.C.A.); flora.deconto@unipr.it (F.D.C.); carlo.chezzi@unipr.it (C.C.); 2 Unit of Clinical Microbiology, University Hospital of Parma, Viale A. Gramsci 14, 43126 Parma, Italy; srossi@ao.pr.it

**Keywords:** intestinal parasitoses, *Dientamoeba fragilis*, gastrointestinal symptoms

## Abstract

*Dientamoeba fragilis* is a cosmopolitan and neglected protozoan. Although little is known concerning its pathogenicity and its true prevalence worldwide, its role as enteric pathogen is emerging, as the occurrence of dientamoebiasis has increased also in industrialised countries. This study investigated the occurrence and prevalence of intestinal parasites, focusing on *D. fragilis* in a 10-year period (2011–2020) in a single tertiary-care hospital located in Northern Italy. A statistical evaluation of the correlation between dientamoebiasis and specific signs other than gastrointestinal-related ones was performed. The laboratory diagnosis was performed on 16,275 cases of suspected intestinal parasitoses. Intestinal parasites were detected in 3254 cases, 606 of which were associated to *D. fragilis*, which represented 18.6% (606/3254) of all the intestinal parasitoses with a 3.7% (606/16,275) prevalence and an increasing trend during the last five years (2011–2015: 2.8% vs. 2016–2020: 4.8%). *D. fragilis* was commonly detected in foreigners, especially those from developing countries, as well as in children; prevalence was equal in males and females. With regard to the clinical aspect, the only putative sign statistically related to dientamoebiasis was anal pruritus. Despite the controversial epidemiological knowledges on dientamoebiasis, the prevalence of *D. fragilis* found in this study highlights the need to consider this parasite in any differential diagnosis of gastrointestinal disease.

## 1. Introduction

*Dientamoeba fragilis* is a protozoan parasite of the human bowel with a worldwide distribution [1,2]. Although it was originally considered as a binucleate amoeba, *D. fragilis* has been reclassified in the family *Dientamoebidae* of the newly revised class *Tritrichomonadidae*, given its antigenic and genetic trichomonad affinities, as well as its flagellate-like features despite the actual lack of flagella [3,4]. Since its first description in 1918 [5], various aspects of the life cycle, the mode of transmission, and the pathogenicity of this parasite have been debated within the scientific community [3,4]. In particular, the potential of *D. fragilis* as a human intestinal pathogen has been questioned for long time, since its mode of nutrition resembles that of the non-pathogenetic amoebas *Entamoeba coli* and *Endolimax nana* [5], and conflicting data has emerged from the earliest few epidemiologic studies in the field. However, the pathogenic role of *D. fragilis* in gastrointestinal illness is strongly supported by several studies accounting the clinical improvement of patients after therapeutic intervention and eradication of the infection [3,4,6,7]. 

It was reported that, patients harbouring *D. fragilis* commonly suffer from diarrhoea, abdominal pain and altered bowel motions, and the clinical spectrum of dientamoebiasis also includes nausea, vomiting, flatus, looseness of stool, presence of blood in faeces, weight loss, and anorexia [3,4,8]. Given its general clinical features, dientamoebiasis is hardly distinguishable from other parasitic bowel diseases, and none of the above-mentioned clinical symptoms can be used as a specific diagnostic criterion for an ongoing *D. fragilis* infection [6,7,8]. 

Interestingly, other clinical signs such as eosinophilia, pruritus, anal pruritus, and urticaria have also been reported in association with dientamoebiasis [9,10,11,12]. In particular, even if peripheral eosinophilia is considered a common clinical sign of any parasitic disease, several case reports have highlighted its characteristic correlation with *D. fragilis* infections, especially in children [9,13,14,15,16,17,18]. On the other hand, few cases of pruritus and urticaria in patients harbouring *D. fragilis* have been reported over the years [12,19,20]. Little is known concerning the correlation between the *D. fragilis* infection and the chronic urticaria, despite that parasitic infections are proven to induce it [21,22]. Furthermore, interesting findings on the pathogenesis of dientamoebiasis came to light due to the development of a rodent model; this also allowed researchers to discover a putative cyst and pre-cyst stage of the life cycle that was unidentified previously. Historically, only the trophozoite stage was known therefore the existence of a vector had been postulated, namely the ova of *Enterobius vermicularis* [3,4]. Such discovery also allowed researchers to corroborate the hypothesis of a faecal–oral transmission [23,24].

Notably, the gastrointestinal pathogenic role of *D. fragilis* is emerging, since both animal- and human-based studies proved its significant association with high levels of faecal calprotectin, a marker of intestinal inflammation [23,25].

With regard to its epidemiology, *D. fragilis* has a cosmopolitan distribution, although it is mostly reported in developed countries, in contrast with the epidemiologic evidence on other intestinal parasitoses [4,7,26,27]. The prevalence rates range between 0.4 and 82%, with the highest incidence figures reported among mental institutions inmates, military personnel, parasitology students, missionaries, and Native Americans in Arizona [3,27,28].

Such prevalence rates and the sex and age distribution of dientamoebiasis tend to vary depending on the geographic area, the cohort studied, and the diagnostic methods employed [3,4,7]. The laboratory diagnosis of *D. fragilis* traditionally relied on microscopy of fixed faecal smears [29], and nowadays it is implemented by more sensitive and specific assays, such as those based on conventional and real-time polymerase chain reaction (RT-PCR) [1,30,31,32,33,34,35,36]. These molecular assays are proving to be accurate and reliable methods for the detection of *D. fragilis* also directly in the stool samples of patients, usually reporting various gastrointestinal complaints [37]. This study aimed to investigate the prevalence rate of dientamoebiasis during a study period of 10 years (2011–2020), and its putative correlation with specific clinical signs other than the gastrointestinal-related ones, in a population attending a tertiary-care hospital located in a non-endemic setting (Parma, Northern Italy). 

## 2. Materials and Methods

### 2.1. Study Design

The study was designed as a retrospective data collection, referred to an observation period of 10 years (2011–2020). Data were sought retrospectively from the records produced by the diagnostic flow of the laboratory where the study was performed, as answers to a clinical suspicion of intestinal parasitosis. 

Laboratory diagnosis was performed upon medical request, and a clinical report was produced. The results of the laboratory diagnosis were analysed in an aggregate form and with anonymized status. Access to patients identification was not possible since anonymisation of samples was conducted before data analysis. For these reasons ethical approval was not required. 

### 2.2. Study Area and Population 

The study was performed at the University Hospital of Parma, a 1104-bed tertiary care centre with more than 39,000 admissions registered in the year 2020 [38]. The province of Parma, located in the Northern Italy, has 452,015 inhabitants, 14% of whom were foreigners, especially those from developing countries [39]; the population attending this hospital was estimated to be 226,130 inhabitants [39].

### 2.3. Patients and Clinical Samples

The laboratory diagnosis was performed on 27,397 faecal samples belonging to 15,624 patients, including both hospitalised subjects and outpatients with suspicion of intestinal parasitosis as reported in the medical order, and sent during the period 2011–2020 to the Parasitology Section of the University Hospital of Parma (Italy) for diagnostic purposes. 

Multiple faecal samples of the same patient were considered as a single case on the basis of the following criteria: (i) all the samples of the same patient analysed for 1 month; (ii) the samples of a patient being always positive for the same parasite continuously over a long time period. On the other hand, when a sample (or a set of samples) of a patient was analysed long after previous specimens were negative or positive for a different parasite, a new case was considered as if it were a new patient with a new suspicion. 

According to these criteria, a total of 16,275 cases of suspected intestinal parasitoses were identified and considered as the same number of patients. 

Among the 16,275 patients, 12,521 were Italians, and 3754 were foreigners especially from developing countries; 3718 were children (split into 3 age groups: 0–5 years, 5–10 years, and 10–18 years) and 12,546 were adults (split into 3 age groups: 18–40 years, 40–60 years, and >60 years), while for 11 cases the age was unknown; 7687 were males and 8588 were females.

The patients’ data concerning the clinical signs and symptoms, such as diarrhoea, abdominal pain, bloody faeces, eosinophilia, pruritus, anal pruritus, urticaria, and/or risk factors such as travels, migration, adoption through/from developing countries, or family history of parasitic infections were obtained from the medical order. 

### 2.4. Diagnosis of Intestinal Parasitosis

The diagnosis of intestinal parasitosis was performed according to standard procedures, as previously described [26]. Briefly, for the detection of parasites, all faecal samples were analysed by macroscopic and by microscopic examination of wet mounts prepared from both fresh and concentrated faeces after formalinethyl acetate sedimentation. An immunocromatographic assay (IC) was performed in order to detect specific antigens of *Cryptosporidium parvum* and *Giardia intestinalis* and positive results by IC were confirmed by an immunofluorescence assay performed as previously described [26].

An additional culture for enteric protozoa in Robinson’s medium and a culture for the larval-stage nematodes according to standard procedures were performed for 10,802 faecal samples, belonging to patients reporting diarrhoea, abdominal pain, bloody faeces, eosinophilia, and/or risk factors for parasitic infections, and/or in whose faeces diagnostic stages of intestinal parasites were detected as previously described [26,32]. 

A real-time PCR assay detecting and differentiating *Entamoeba histolytica* and *Entamoeba dispar* was performed as previously described [26].

Moreover, in particular, 1457 out of 10,802 faecal samples subjected to culture were also investigated by a TaqMan real-time PCR assay for *D. fragilis*, as previously described [24,26,32].

### 2.5. Statistical Analysis 

Demographic data (origin, age, and sex) collected was related to the detected parasitic infections. The statistical significance of such data in association with intestinal parasitic infections into each demographic group was calculated by a *chi*-square test: a *p* value < 0.05, calculated by two-tailed test, was considered significant. Odds ratios (ORs) were calculated in order to evaluate the strength of the associations which emerged.

## 3. Results

Intestinal parasites were detected in 3254 out of 16,275 suspected cases, corresponding to a prevalence rate of 20%. A statistical analysis concerning the origin, age, and sex of the patients diagnosed with intestinal parasitosis is reported in Table 1.

For each demographic group, intestinal parasitosis involved 15.3% (1910/12,521) of Italians and 35.8% (1344/3754) of foreigners, especially those from developing countries (*p* < 0.00001; OR = 0.32), as well as 18.3% (679/3718) of children and 20.5% (2575/12,546) of adults (*p* = 0.0025; OR = 0.85), and both rates spread among the different respective age groups as shown in Table 1; intestinal parasitosis involved 23.6% (1817/7687) of males and 16.7% (1437/8588) of females (*p* < 0.00001; OR = 1.54) (Table 1). 

A total of 4193 intestinal parasites was detected, 92.1% (3863/4193) of which were protozoa and 7.9% (330/4193) were helminths. Among the total parasitic infections diagnosed, 75.2% (2448/3254) were caused by a single parasite (protozoan, 2308 cases, or helminth, 140 cases), and 24.8% (806/3254) were associated with more than one parasite (at least two protozoa, 635 cases; or helminths, 7 cases; rather than a combined protozoan–helminth infection, 164 cases) (Table S1). 

**Figure 1:**
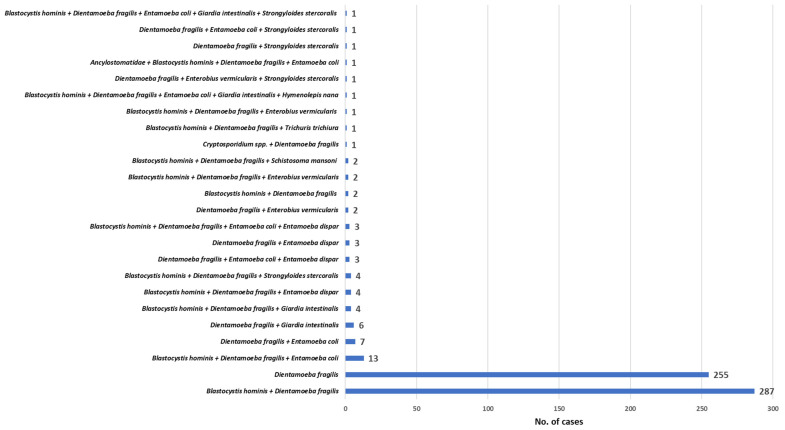
Different combinations of the 606 cases of dientamoebiasis.

On the overall, *D. fragilis* was detected in 606 cases (Table S2), representing 18.6% (606/3254) of all the intestinal parasitoses and 14.4% (606/4193) of all the intestinal parasites detected, with a prevalence rate of 3.7% (606/16,275). In 255 out of 606 cases (42.1%; 255/606), *D. fragilis* was the only parasite detected, while in the remaining 351 cases (57.9%; 351/606), it was found in combination with other protozoa (94.9%; 333/351), helminths (1.1%; 4/351), or both protozoa and helminths (4%; 14/351), as summarised in Figure 1. 

**Figure 2:**
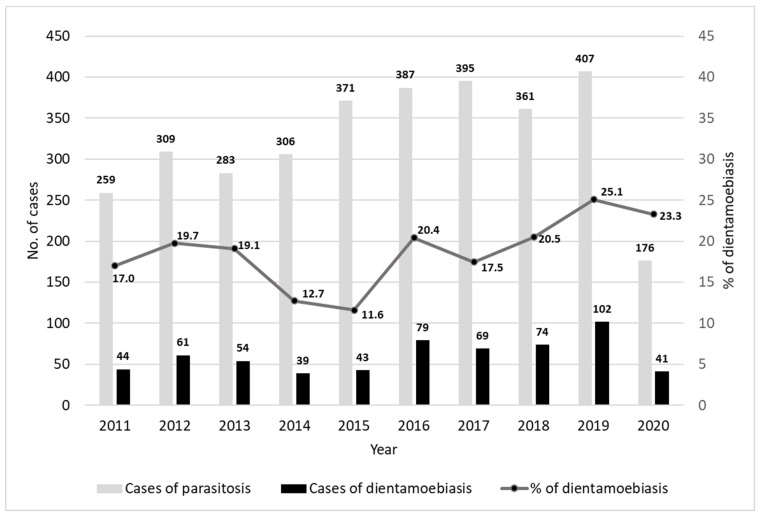
Distribution of cases and frequency of dientamoebiasis per year.

The cases of dientamoebiasis in comparison with total intestinal parasitoses per each year are reported in Figure 2.

The frequency of *D. fragilis* ranged from 16% in the period 2011–2015 to 21.36% in the period 2016–2020, accounting for an average of 60 cases/year.

The prevalence of dientamoebiasis showed an increasing trend during the last five years: 2.8% (241/8611) was reported in 2011–2015 vs. 4.8% (365/7664) during 2016–2020. 

A statistical analysis concerning the origin, age, and sex of the patients diagnosed with dientamoebiasis is reported in Table 2.

For each demographic group, dientamoebiasis involved 3.2% (394/12,521) of Italians and 5.7% (212/3754) of foreigners, especially those from developing countries (*p* < 0.00001; OR = 0.54). In 5.4% (200/3718) and 3.2% (406/12,546), *D. fragilis* infected children and adults, respectively (*p* < 0.00001; OR = 1.70), and both rates spread among the different respective age groups as shown in Table 2; 3.7% (283/7687) of males and 3.8% (323/8588) of females (*p* = 0.7891; OR = 0.98) were infected by *D. fragilis* (Table 2). 

The rates of detection of *D. fragilis* by each single diagnostic method (microscopic examination, cultural method, and nucleic acid amplification assay by real-time PCR) are reported in Table 3. Microscopy examination allowed to identify *D. fragilis* in the 56.8% of cases in combination with real-time PCR or with real-time PCR and culture; on the other hand, in the 9.1% of cases, *D. fragilis* was detected only by the molecular method.

As concern the clinical aspects, for 1616 out of the 3254 positive cases, signs and/or symptoms were reported in the medical order. In 699 cases (43.3%; 699/1616) only gastrointestinal-related signs and symptoms such as diarrhoea, abdominal pain, and bloody faeces were reported in the medical order; in 171 cases (10.6%; 171/1616), gastrointestinal-related signs/symptoms were reported in association with signs and symptoms other than the gastrointestinal-related ones; in the remaining 746 cases (46.2%; 746/1616), only signs/symptoms other than the gastrointestinal-related ones were indicated.

Overall, among the 937 (171 + 746) cases with signs and symptoms other than the gastrointestinal-related ones, eosinophilia (40.3%; 378/937), pruritus (17.5%; 164/937), urticaria (8.8%; 82/937), anal pruritus (5.1%; 48/937), and eczema/dermatitis (2.9%; 27/937) were the most frequently reported.

With regard to dientamoebiasis, for 287 out of 606 cases, signs and/or symptoms were reported in the medical order. In particular, in 132 cases (46%; 132/287), only gastrointestinal-related signs/symptoms were reported; 22 cases (7.7%; 22/287) were in association with signs/symptoms other than the gastrointestinal-related ones; in the remaining 133 cases (46.3%; 133/287), only signs/symptoms other than the gastrointestinal-related ones were indicated. 

The statistical analysis concerning the association between the most frequent clinical signs other than the gastrointestinal-related ones and dientamoebiasis for each demographic group is reported in Table 4. 

The putative correlation of such clinical signs with dientamoebiasis was evaluated: the occurrence of pruritus, urticaria, and eczema/dermatitis was not statistically different (*p* = 0.3738 OR= 0.82; *p* = 0.1882 OR = 0.42; *p* = 0.2629 OR= 1.64, respectively) in cases of dientamoebiasis or other intestinal parasitoses. On the other hand, anal pruritus (*p* < 0.00001 OR = 4.54) and eosinophilia (*p* = 0.0012 OR = 0.57) were shown to be statistically correlated to dientamoebiasis and the other intestinal parasitoses detected, respectively.

In particular, in 10 out of the 23 cases of dientamoebiasis reporting anal pruritus, *D. fragilis* was the only parasite detected, while the remaining 13 were in association with at least one protozoan (10 with *Blastocystis hominis*, 1 with *Entamoeba dispar*, 1 with *B. hominis* and *E. dispar*, and 1 with *B. hominis* and *Entamoeba coli*). In 5 out of the 23 cases associated to anal pruritus, the scotch test for the detection of *E. vermicularis* was performed with a negative result.

In the 606 cases of dientamoebiasis, a follow up was performed in 209 cases, and in 75 cases (12.4%; 75/606), an indication of therapy was reported in the medical order. Of these latter cases, 13 (17.3%; 13/75) resolved signs and symptoms after therapy and a *D. fragilis*-negative sample was found; for the remaining 62 cases the last sample examined was still positive for *D. fragilis.*

## 4. Discussion

As concern the epidemiological aspects, little is known about the geographic and demographic distribution of dientamoebiasis in the industrialised countries [7,27,33,34]. This study evaluated the occurrence and prevalence rate of intestinal parasites focusing on *D. fragilis* in a 10-year period (2011–2020) in a single tertiary-care hospital located in Northern Italy.

The high prevalence of intestinal parasitic infections detected in this study (20%) had increased when compared to the one found in a previous study (16.6%) conducted in the same area during the five previous years (2006–2010) [26], and unexpectedly in a high-level health standards area. This evidence could be due to the high number of patients’ samples from developing countries (immigrates or travelers from) and therefore presenting risk factors for acquiring parasitic infections. As a matter of fact, only 15.3% of Italians patients presented a parasitosis, against 35.8% of foreigners (*p* < 0.00001 OR = 0.32). Moreover, the parasitoses were significantly more common in males than in females (*p* < 0.00001 OR = 1.54) and in adults than in children (*p* = 0.0025 OR = 0.85), in line with the results of the previous study [26].

With regards to aetiology, helminthic infections confirmed to be less frequent than the protozoan ones (7.9% vs. 92.1% on the total of detected parasites). In addition, single-agent infections showed to be prevalent than mixed infections (75.2% vs. 24.8%, respectively). As concern the mixed infections, 78.8% were referred to at least two protozoa, 20.3% were a combined protozoon–helminth infection, and 0.9% were caused by at least two helminths.

In contrast to our previous results [26], *D. fragilis* showed an year-by-year increasing trend of both rates of frequency (18.6% vs. 8.3%) and prevalence (3.7% vs. 1.68%). Furthermore, the demographic distribution of dientamoebiasis thoroughly changed at ever-increasing rates: the previous study period accounted for 30 cases/year (149 total cases of dientamoebiasis) against the 60 cases/year (606 total cases of dientamoebiasis) reported in this study, with a doubling trend. 

Notably, the frequency of *D. fragilis* ranged from 16% in the period 2011–2015 to 21.36% in the period 2016–2020 with a medium increase rate of 5% and reaching the highest peak in 2019 (25.1%). This increasing trend of both these frequency and prevalence rates could be related to the more frequent use of molecular methods combined with conventional ones. In agreement with these considerations, also Intra et al. [29] suggested that the implementation of the conventional method with other diagnostic techniques could significantly affect the rates of detection, with a positive trend reported. Since the microscopic evaluation of faecal smears largely depends on microscopists’ experience, the real rates of distribution of *D. fragilis* could be underestimated. Therefore, the lack of accurate and sensitive diagnostic methods, such as molecular ones, could lead to an under-evaluation of the real rates of frequency among the population [21,29]. 

New diagnostic approaches, such as the MALDI-TOF mass spectrometry, already successfully used for the identification of this protozoan, could fill this gap [40]. 

Despite the few faecal samples tested during the COVID-19 pandemic period in 2020, the rates of prevalence of *D. fragilis* were maintained at high rates, in line with the increasing trend, reporting the second high peak (23.3%) of the last ten years.

In this study, dientamoebiasis was significantly more frequent in foreigners than in Italian patients (*p* < 0.00001 OR = 0.54) and in children than in adult patients (*p* < 0.00001 OR = 1.70); no statistically significant differences were found between males and females (*p* = 0.7891 OR = 0.98). Although an overall increase emerged from the statistical analysis concerning the demographic distribution of dientamoebiasis during the 2011–2020 period, all these results are in agreement with those reported in the previous study [26]. 

*D. fragilis* was the only parasite detected in 255 cases (42.1%) and in 351 cases (57.9%) was in association with other parasites, for a total of 23 different combinations, most frequently with *B. hominis* (81.8%; 287/351). This combination is not unexpected, since in both this and previous studies, *B. hominis* was often reported as the most commonly detected organism in parasitological surveys [6,26,41]. 

Concerning the clinical aspects, 53.7% of dientamoebiasis were associated to gastrointestinal-related signs and symptoms, while the remaining 46.3% reported signs and symptoms other than the gastrointestinal-related ones, among which eosinophilia, pruritus, urticaria, anal pruritus, and eczema/dermatitis were the most reported. Despite a statistically significant correlation was found for anal pruritus, few cases (23) reported such sign, and, moreover, 13 of whom were associated to a mixed infection with at least one protozoan, especially *B. hominis* (12 cases). In addition, only in 5 out of these 23 cases *E. vermicularis* was searched for with a specific test and excluded as a cause of anal pruritus.

While in cases of mixed infection the role/contribution of *D. fragilis* to the observed symptoms may not be clear, in cases of symptomatic patients with a documented absence of enteropathogenic parasites, bacteria and/or viruses, *D. fragilis* may be considered the causative agent of the symptoms. In this study, only in 3 out of the 255 cases associated to *D. fragilis* as unique parasite another enteropathogenic agent was revealed (Rotavirus, Norovirus, and enteroaggregative *Escherichia coli*, respectively) (data not shown). 

Concerning the treatment of the patients, any follow up was not performed in more than 65% of the cases, thus no other information regarding the outcome was available, and only in 12.4% of cases of dientamoebiasis an indication of therapy was reported in the medical order; overall, for only 2.1% of cases related to *D. fragilis,* a negativization following therapy was observed. Given the limited information available on the medical order, it is not possible to establish a real correlation between the infection and therapy, signs, symptoms, and outcome. However, it could be speculate that patients with no follow-up had no longer signs/symptoms.

## 5. Conclusions

In this study *D. fragilis* was found to be the second most detected protozoan after *B. hominis* and more than two times as prevalent than *Giardia intestinalis*.

Despite the controversial epidemiological knowledge on dientamoebiasis, an underestimation of the real prevalence rates is highly probable due to the intrinsic difficulties in detection and identification [6,32], and the true incidence of this infection could be higher than reported. As a matter of fact, in this study, the prevalence of *D. fragilis* was found to be 3.7%, significantly higher than that reported in the same area in a previous period (1.68%), highlighting the common occurrence of this protozoan parasite that should be considered in any differential diagnosis of gastrointestinal disease.

Concerning the clinical evaluation conducted in this study, no statistical correlation with dientamoebiasis was found, except for anal pruritus, which could be the starting point for further investigations in the field in order to confirm its presumptive association with *D. fragilis*, and to better define any possible association with other specific clinical signs and symptoms.

## Figures and Tables

**Table 1:** Statistical analysis concerning the origin, age, and sex of the patients diagnosed with intestinal parasitosis.

			Total Patients (No.)	Positive Patients (No.)	Intestinal Parasitoses (%)	Odds Ratio	*p* Value
ORIGIN	Italians		12,521	1910	15.3	0.32	<0.00001
	Foreigners		3754	1344	35.8		
AGE	Children		3718	679	18.3	0.85	0.0025
	Adults	0–5	1356	177	13.1		
		5–10	1293	289	22.4		
		10–18	1069	213	19.9		
			12,546	2575	20.5		
		18–40	4692	1185	25.3		
		40–60	4020	815	20.3		
		>60	3834	575	15		
	Unknown		11	0	0		
SEX	Males		7687	1817	23.6	1.54	<0.00001
	Females		8588	1437	16.7		

**Table 2:** Statistical analysis concerning the origin, age, and sex of the patients diagnosed with dientamoebiasis.

			Total Patients (No.)	Positive Patients (No.)	Dientamoebiasis (%)	Odds Ratio	*p* Value
ORIGIN	Italians		12,521	394	3.2	0.54	<0.00001
	Foreigners		3754	212	5.7		
AGE	Children		3718	200	5.4	1.70	<0.00001
		0–5	1356	36	2.7		
		5–10	1293	109	8.4		
		10–18	1069	55	5.2		
	Adults		12,546	406	3.2		
		18–40	4692	148	3.2		
		40–60	4020	177	4.4		
		>60	3834	81	2.1		
SEX	Males		7687	283	3.7	0.98	0.7891
	Females		8588	323	3.8		

**Table 3:** Detection of *D. fragilis* by each single diagnostic method.

Diagnostic Method	No. of Cases (%)
Culture + real-time PCR	207 (34.1)
Microscopic examination + real-time PCR	180 (29.7)
Microscopic examination + Culture + real-time PCR	164 (27.1)
real-time PCR	55 (9.1)
Total	606

**Table 4:** Statistical analysis concerning the association between the most frequent clinical signs other than the gastrointestinal-related ones and dientamoebiasis for each demographic group.

			Eosinophilia (46 Cases)	Pruritus (25 Cases)	Urticaria (19 Cases)	Anal Pruritus (23 Cases)	Eczema/Dermatitis (7 Cases)
ORIGIN	Italians		25	14	13	15	5
	Foreigners		21	11	6	8	2
AGE	Children		20	1	2	7	0
		0–5	4	1	0	2	0
		5–10	9	0	2	2	0
		10–18	7	0	0	3	0
	Adults		26	24	17	16	7
		18–40	13	9	7	4	2
		40–60	5	13	8	11	3
		>60	8	2	2	1	2
SEX	Males		30	10	8	13	2
	Females		16	15	11	10	5

## Data Availability

The data presented in this study are available in the manuscript and in the Supplementary Materials (Tables S1 and S2).

## Supplementary Materials

The following supporting information can be downloaded at: https://www.mdpi.com/article/10.3390/microorganisms10020426/s1, Table S1: cases of intestinal parasitosis detected during the study period; Table S2: cases of dientamoebiasis detected during the study period.
